# The Impact of Being Homeless on the Clinical Outcomes of COVID-19: Systematic Review

**DOI:** 10.3389/ijph.2023.1605893

**Published:** 2023-09-15

**Authors:** Obianuju Ogbonna, Francesca Bull, Bethany Spinks, Denitza Williams, Ruth Lewis, Adrian Edwards

**Affiliations:** ^1^ Health and Care Research Wales Evidence Centre, Division of Population Medicine, School of Medicine, Cardiff University, Cardiff, United Kingdom; ^2^ Division of Population Medicine, School of Medicine, Cardiff University, Cardiff, United Kingdom; ^3^ North Wales Centre for Primary Care Research, School of Medical and Health Sciences, Bangor University, Bangor, United Kingdom

**Keywords:** homelessness, COVID-19 pandemic, systematic review, public health impacts, public health

## Abstract

**Objective:** The homeless population experiences inequality in health compared with the general population, which may have widened during the COVID-19 pandemic. However, the impact of being homeless on the outcomes of COVID-19 is uncertain. This systematic review aimed to analyse the impact of experiencing homelessness on the clinical outcomes of COVID-19, including the effects on health inequalities.

**Methods:** A review protocol was developed and registered in PROSPERO (PROSPERO registration 2022 CRD42022304941). Nine databases were searched in November 2022 to identify studies on homeless populations which contained primary research on the following outcomes of COVID-19: incidence, hospitalisation, mortality, long COVID, mental wellbeing, and evidence of inequalities. Included studies were summarised with narrative synthesis.

**Results:** The searches yielded 8,233 initial hits; after screening, 41 studies were included. Overall, evidence showed that those in crowded living settings had a higher risk of COVID-19 infection compared to rough sleepers and the general population. The homeless population had higher rates of hospitalisation and mortality than the general population, lower vaccination rates, and suffered negative mental health impacts.

**Conclusion:** This systematic review shows the homeless population is more susceptible to COVID-19 outcomes. Further research is needed to determine the actual impact of the pandemic on this population, and of interventions to mitigate overall risk, given the low certainty of findings from some of the low-quality evidence available. In addition, further research is required to ascertain the impact of long COVID on those experiencing homelessness, since the present review yielded no studies on this topic.

## Introduction

### Background

People experiencing homelessness also experience inequality in health compared to the general population [1, 2], and the COVID-19 pandemic is thought to have widened health inequalities [3]. However, we do not know the overall impact of being homeless on the clinical outcomes of COVID-19, and on the pre-existing health inequalities of this marginalised group.

Pre-existing health inequalities are one aspect of why people experiencing homelessness may have been disadvantaged regarding COVID-19, and there has been concern for this population since the onset of the pandemic [4]. A homeless person is more likely to: have a long-term condition [5]; contract, spread, and die from viruses such as influenza or HIV [6, 7]; and have difficulty accessing healthcare and following national guidance [8].

It is thought that homelessness itself directly impacts health through issues such as extreme temperatures, malnutrition, and lack of amenities for personal hygiene [1, 9]. Additionally, there is perceived stigma and judgement from much of the general population, and feelings of isolation which can play into worse psychosocial wellbeing [10, 11].

Both people from more deprived groups and those with long-term conditions have increased risks of negative outcomes of COVID-19 [12]. Much research also examines the negative impact of the COVID-19 pandemic on people’s mental health [13]. Given that a person experiencing homelessness is more likely to be socially deprived, have a long-term condition or worse physical condition [3], or struggle with mental illness [14, 15], this population may have been at higher risk of negative impacts of the COVID-19 pandemic.

Additionally, the homeless population is marginalised from society and may not have had the same attitudes and access as the general population to mediators of COVID-19 outcomes. These include the availability of testing and vaccination, adoption of personal protective behaviours, and access to healthcare.

One US-focused systematic review assessed COVID-19 prevalence, transmission, and control strategies in homeless shelters [16]. The evidence in this review is important, but less relevant to the settings such as the UK where there has been a shift away from use of homeless shelters [17]. Babando et al. also summarised literature on disease outbreaks and pandemic responses among people experiencing homelessness, with recommendations that were not in-depth on the COVID-19 pandemic specifically, countries, or subtypes of homeless populations [6]. There has not yet been a systematic review examining the outcomes as well as mediators of COVID-19 in the homeless population.

### Aim and Objectives

We aimed to review the impact of experiencing homelessness on the clinical outcomes of COVID-19. To do this, our objectives were: to describe the outcomes of COVID-19 in the homeless population; to examine whether health inequalities between homeless and general populations have widened during the pandemic; to investigate the reasons for severe impact or widened inequalities (if identified) in relation to COVID-19 management-related mediators and homelessness risk factors.

## Methods

A review protocol was developed and registered in PROSPERO (PROSPERO registration 2022 CRD42022304941). This systematic review was conducted following the good practice guidelines [18], and reporting was guided by the standards of the PRISMA Statement [19].

### Selection Criteria

The selection criteria for this review were determined by relevant literature and in consultation with key stakeholders who are experts in research and public health concerning people experiencing homelessness (Table 1).

**TABLE 1:** Eligibility criteria for selecting studies. Wales, United Kingdom, 2023.

	Inclusion criteria	Exclusion criteria
Population	People aged 16 and over experiencing homelessness during COVID-19 pandemic	No research on homeless population
	Using the ETHOS definition of homelessness, see Supplementary Table S1 [20]	People under the age of 16
Exposure	COVID-19 outbreak	Other previous pandemics/diseases
Comparisons	i. Describing the outcomes of COVID-19 in the homeless population and comparing them to the general population, to other marginalised populations, between subtypes of homelessness, or over time	No comparison
	ii. Describing the inequalities experienced by the homeless population and comparing them with before the COVID-19 pandemic	
Outcome measures	Studies on **clinical outcomes** related to COVID-19: rates of COVID-19 infection, hospitalisation rates, mortality rates, mental health impact, and long COVID rates	Clinical outcomes of COVID-19 not measured
	Studies looking at **mediators** which affect COVID-19 outcomes, including but not limited to attitudes to COVID-19, testing rates, vaccination uptake, adoption of personal protective behaviours, and accessibility to healthcare/other services	
	Studies reporting on the impact of the COVID-19 pandemic on wider health and socioeconomic **inequalities**	
Study design	Hierarchy of evidence, prioritising primary research studies with a comparison. Both quantitative and qualitative studies were reviewed. Grey literature considered if providing empirical data	Opinion pieces without empirical data
		Systematic reviews (References lists checked)
Language	English	Not published in English
Publication date, type	During/Since start of pandemic, published and preprint	Prior to 2020

### Search Strategy

The search strategy was developed in consultation with a subject librarian. To reduce complexity and achieve high recall, the search strategy used two key concepts: COVID-19 and homelessness (combined with AND). The search string for COVID-19 was developed by systematic review experts (Supplementary Appendix SA). The string for homelessness was adapted from published systematic review searches [5, 16], in line with the ETHOS definition [20]. Further details of the abbreviated ETHOS framework of homelessness and housing exclusion can be found in Supplementary Appendix SB.

The search strategy was run on MEDLINE (OVID) then adapted for use on the following databases: Embase, CINAHL, Cochrane Library, ASSIA, Web of Science, L*VE Evidence, Social Policy and Practice, and Scopus Embase and L*VE Evidence include preprints of which final publications were sought if available. Relevant reference lists were checked and resources proposed by stakeholders were considered. The searches were conducted on 28 November 2022.

### Study Screening and Selection

The results from each database were exported to the reference management software EndNote [21], de-duplicated, and screened for eligibility applying the criteria (Table 1). Studies published before 2020 or not in English were excluded after de-duplication, rather than using database search filters, to prevent errors in retrieving relevant articles.

Titles and abstracts were screened (by FB or OO) and 10% of the results (randomly selected) were screened by another team member (BS) to ensure consistency in using the eligibility criteria. There were few disagreements (approximately 1% of the peer-screened selection) which were discussed and resolved. Involvement of a third reviewer was not necessary as the differences in selection were minimal. Full text analysis of the remaining potential studies was conducted by a single reviewer (FB or OO).

It was anticipated that there would be a low number of robust, high-quality eligible studies for this systematic review. Therefore, instead of excluding lower-level evidence sources, a hierarchy of evidence was used following the Evidence-based Medicine Pyramid study design [22].

### Data Extraction

Microsoft Excel was used to extract data from the included studies: authors and title, setting, population, study design and methodology, study period, outcomes (or mediators), results, and limitations as reported in the study (abridged version Supplementary Table S1, *Results* section).

### Quality Assessment

Internal validity was assessed through critical appraisal by a single reviewer (FB or OO) using the appropriate Joanna Briggs Institute (JBI) Checklist for study design, including assessing issues such as methodological approach, missing data, and low response rates or sample size [23–25]. External validity was assessed in respect to transferability to other settings for people experiencing homelessness. The conclusions of these assessments were combined with consideration of the study design based on the evidence pyramid [22] to provide an overall assessment of the quality of the study.

### Synthesis

Narrative synthesis was performed, structured using a framework analysis [26] with the following headings:

• Incidence of COVID-19 infection
• Hospitalisation from COVID-19 infection
• Mortality from COVID-19
• Mental wellbeing impact of COVID-19
• Long COVID rates
• Mediators of COVID-19, including vaccination rates
• Health inequalities during the COVID-19 pandemic.

Meta-analysis was not feasible due to the heterogeneity of study designs, populations studied, and evaluation of outcomes.

Analysis of subgroups was intended if studies focused on or specified between particular subtypes of homelessness (e.g., using ETHOS categories).

## Results

### Selection of Included Studies

**FIGURE 1:**
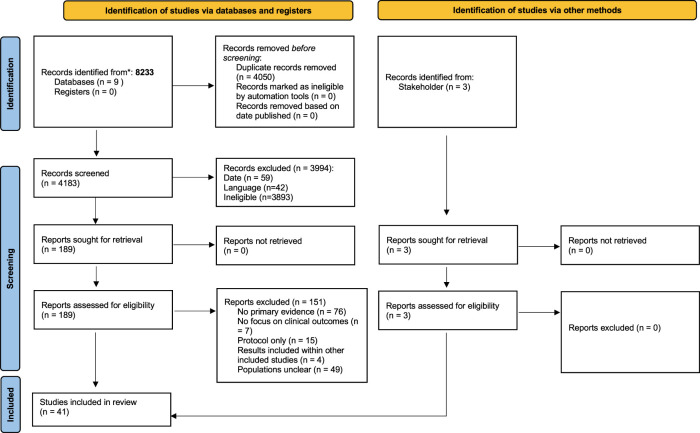
Flow diagram of study selection process. Wales, United Kingdom, 2023.

From 8,233 initial hits from database searches, 4,183 references remained after de-duplication. A total of 181 studies published before 2020 were removed. After title and abstract screening, 189 articles remained for full text analysis, 38 of which were eligible for this review. Three studies, published after the search was conducted, were proposed by stakeholders. A total of 41 studies were included in this systematic review (Figure 1) [27–67].

Supplementary Table S1 details the included studies, which were descriptive or observational in design. The most common country of origin was the United States [29–31, 35–37, 39, 40, 43, 46, 47, 50, 59, 62, 67], then France [27, 32, 33, 38, 41, 42, 44, 45, 52, 56, 60], with further contributions from Wales [63, 65, 66] and Denmark [34, 48, 49, 64].

Most studies defined the type of homeless population researched to some degree (e.g., rough sleepers or those in hotel accommodation). Four did not include any specification of populations, and most were vague in how they determined homelessness type. Comparators included the general population and shelter workers; comparisons were also made between homelessness types and time periods of the pandemic. Comparisons were often at high risk of bias relating to study design or unclear in reporting of methodological approach.

The overall quality of studies was moderate, with 21 of the 41 studies rated as such by critical appraisal. However, the quality of 30% of studies was low due to issues in methodological approach, missing data, and low response rates or sample size.

In the following sections, when reporting the results of the included studies, the term significant is used to describe statistical significance.

### Incidence of COVID-19 in People Experiencing Homelessness

Nineteen of the included studies reported on the prevalence of COVID-19 in the homeless population in varying detail [27, 34, 38, 39, 42–45, 47, 51, 52, 54, 56–59, 63–65]. The quality of the evidence was mostly low to moderate, with three studies of high quality [54, 63, 67].

Seven studies found higher rates of COVID-19 infection among people experiencing homelessness compared to the general population [34, 42, 56, 63] and workers or volunteers associated with the homeless population studied [38, 44, 54]. One study compared rates of COVID-19 between those staying in homeless shelters versus outdoor encampments and found high rates of infection in the latter group [59]. One study found similar rates to its background population [64], and two reported lower rates of COVID-19 infection among people experiencing homelessness [47, 65]. When accounting for demographic confounding factors, one study detected no difference in the incidence of COVID-19 in unhoused patients attending an emergency department as compared with their housed counterparts [39].

Some studies compared between subgroups of people experiencing homelessness based on ETHOS categories or crowding levels. Shelters and overcrowding were associated with increased risk of COVID-19 infection and outbreak clusters compared to less crowded accommodation [38, 42, 44, 56, 57, 59], rough sleepers [27, 38], and food distribution sites [56]. Four studies that reported on COVID-19 prevalence did not have valid comparisons to interpret rates [44, 51, 52, 58].

Risk factors beyond living conditions, such as gender, age, and comorbidities, were reported in some studies. Three studies found that gender was not a significant risk factor for COVID-19 infection, while one study found that being female was associated with reduced risk. Neither age [42] nor comorbidities [57] were found to have significance in terms of infection risk. One study found people who were homeless and obese had an increased risk of seropositivity of COVID-19 [34]. One study cited race, age, and living in outdoor encampments as factors increasing the likelihood of COVID-19 among those experiencing homelessness [59].

### Hospitalisation Rates Due to COVID-19 Infection in People Experiencing Homelessness

Seven studies reported hospitalisation due to COVID-19 infection in the homeless population [30, 38, 40, 48, 52, 61, 63].

People experiencing homelessness had significantly greater risk of hospitalisation due to COVID-19 infection compared to the general population [30, 38, 40, 48, 61, 63]. Reporting on risk varied among these studies. Of the two high-quality studies, one calculated an incidence rate ratio (IRR) of 2.1 for hospitalisation of a homeless person [48], and one reported the hospitalisation rate was 9 per 1,000 in the homeless population and 1 per 1,000 in the general population of Wales [63].

Comorbidities were found to increase risk of hospitalisation of people experiencing homelessness [38, 61], although it was noted that comorbidities were generally common in all hospital patients. Hospitalised people experiencing homelessness had higher rates of alcohol and drug misuse compared with the general population patients [61].

Differing findings were seen regarding intensive care unit (ICU) admission. Two cohort studies with robust methodological approaches found rates of ICU admission higher proportionally in people experiencing homelessness compared with the general population (non-peer-reviewed publication) [48]. Another study, of low quality, found no significant difference in ICU admissions [61].

One study found a higher proportion of patients hospitalised with COVID-19 were from insecure living conditions during lockdown compared to pre-lockdown [52].

No studies reported on how types of homelessness impacted risk of hospitalisation.

### Mortality Rates Due to COVID-19 Infection in People Experiencing Homelessness

Five studies reviewed mortality due to COVID-19 infection. All found higher mortality rates in the homeless population compared with the general population [30, 31, 38, 48, 63].

A study looking at people experiencing homelessness in Wales reported COVID-19 mortality rates three times that of the general population [63]. In Denmark, the mortality rate ratio compared with the general population was 3.2 (30 day mortality after COVID-19 infection) and 5.6 (all-cause mortality) [34]. This study found that a positive PCR test was associated with a four-times increase in mortality for people experiencing homelessness. Having adjusted for race and sex, one study in Los Angeles calculated standardised mortality ratios among the homeless population of 1.4 and 1.2, respectively [31].

Studies reporting on mortality did not find significant evidence on other risk factors but did report on general high rates of health problems. Other risk factors for incidence are as noted in *Incidence of COVID-19 in People Experiencing Homelessness* section.

### Mental Wellbeing Impact Associated With COVID-19 Pandemic

Eight studies of varying quality reported on the mental wellbeing of homeless populations during the COVID-19 pandemic.

Two studies found that people experiencing homelessness reported feelings of loneliness or isolation during the COVID-19 pandemic [28, 58]. Risk factors for loneliness included being male, living in crowded accommodation, and self-perceived risk of COVID-19 [28]. The authors note that the finding of increased feelings of loneliness in more crowded accommodation reflects a theory in literature that self-perceived social isolation is independent of the amount of social interaction [68]. The proportion of people experiencing homelessness feeling lonely was compared to the findings of a 2012 survey of the background population; this comparator therefore did not fully consider the impact of COVID-19 [28].

Another study looked at how lockdown impacted the young people experiencing homelessness in Wales, and found lockdown improved mental wellbeing, self-esteem, and physical activity [65]. However, these outcomes were still far below the national average.

Five studies adopted a qualitative methodology and examined the personal impact of the pandemic on people experiencing homelessness [32, 35, 36] as well as those working in homeless shelters [37, 50]. Several studies described deleterious impacts of the pandemic on mental health [36, 50], citing worries about COVID-19 and limited accessibility to substance-misuse support because of the pandemic. In other studies, participants described feeling restricted in their ability to manage their personal health due to a reduced capacity to isolate effectively [32], challenges in dealing with uncertainty, and a perception of restrictions on normal activities [32, 37]. Two studies mentioned increased drug use, alcohol use, and smoking as coping mechanisms [36, 50]. One study found people were more concerned about financial-rather than health-related repercussions of the pandemic [35].

One study reported on deaths in the homeless population during the pandemic due to non-COVID-19 causes and found twice as many deaths in the first year of the pandemic as compared with the preceding years [29]. Deaths were most attributed to trauma and substance misuse [29].

A cross-sectional study reviewed depression among people experiencing homelessness in France [60]. Of 527 subjects, 30% displayed features of moderate to severe depression [60]. The study identified the following risk factors for depression within this population: chronic illness, female gender, singlehood, uncertain access to food, age between 18 and 29, and finally, migration from African and Eastern Mediterranean countries rather than other European countries [60].

### Long COVID in People Experiencing Homelessness

There were no studies identified from the search reporting on long COVID in people experiencing homelessness.

### Mediators to COVID-19 in People Experiencing Homelessness

Mediators to COVID-19 were defined as factors that could directly or indirectly contribute to the risk of COVID-19 outcomes. This included attitudes to the pandemic, adherence to public health measures, and access to healthcare (see Table 1).

Three studies looked at mediators to COVID-19 in people experiencing homelessness, mostly focusing on attitudes and adherence to COVID-19 preventive measures, and adoption of behaviours such as social distancing and mask-wearing. Measuring rates of adherence was often done through self-reporting so these studies have limited reliability.

One study found reported adherence with measures was high [27], although there were conflicting results between quantitative and qualitative findings. In interviews, people experiencing homelessness reported challenges complying with social distancing measures when living in accommodations such as shelters. Another study found rates of adherence to be fairly high but still lower than the rates in workers in the homeless accommodation being studied [34]. A low-quality study found that access to hygiene materials for people experiencing homelessness improved in the second lockdown compared with the first, and self-reported adherence to measures was very high [58].

Six studies explored the trends of COVID-19 vaccine uptake. Two were high quality [49, 60] and four were moderate quality [33, 45, 55, 67]. Overall, the studies found that people experiencing homelessness were less likely to have received both doses of the vaccine than their housed counterparts. Of the high-quality studies, one calculated a risk ratio of 0.5 for vaccine uptake in people experiencing homelessness, after accounting for age and time of year [49]. The other study determined that residence in a smaller more rural area was linked with reduced uptake compared with a larger more urban area [62]. Furthermore, those who had received the flu vaccine in a prior flu period, with more than one chronic health issue (adjusted risk ratio 1.11), and had visited the GP on at least one occasion (adjusted risk ratio 1.37) were more likely to have had the COVID vaccine [62].

Concerning the moderate quality studies, one found a reduced vaccination frequency in young adults who had recently experienced homelessness, as compared with the vaccination rates of the general population [67]. Across the studies, reasons cited for vaccine reluctance were as follows: poor accessibility to vaccines, wariness about the long-term effects, and fears about vaccine efficacy [33, 46]. One study found that vaccine uptake was positively correlated with age and flu vaccine receipt and negatively linked with Black race and female sex [55]. Approximately 80% of the 728 respondents had received at least one dose [55].

One cross-sectional study examined attitudes toward vaccination among those experiencing homelessness in France [41]. The percentage of participants expressing reluctance towards vaccine uptake (approximately 41%) was in keeping with that of the wider population [41]. The study cited female gender, cohabitation, poor health literacy, and French citizenship as factors reducing the likelihood of vaccine uptake within this population [41].

### Health Inequality

While all included studies looking at this marginalised group are reporting on potential inequalities, two of the included studies identified specific examples connected to the COVID-19 pandemic.

One study reported that 25% of the sample population had unmet healthcare needs during lockdown, as well as many having severe financial issues and lack of access to primary care resources, especially for rough sleepers [27]. Rates of emergency department (ED) attendance and emergency admissions in people experiencing homelessness were also significantly higher than the general population, likely highlighting unmet healthcare needs and issues with primary care provision [63]. Overall, our study highlights a paucity of research on health inequalities within homeless populations.

## Discussion

### Summary of Principal Findings

This systematic review shows evidence of differences in clinical outcomes for people experiencing homelessness compared with the general population or those who work with them, as well as between subgroups of the homeless population. Homeless populations in crowded environments such as shelters and hotel accommodations were at higher risk of COVID-19 infection, especially compared with those sleeping rough, although the evidence was of limited quality [27, 38, 42, 44, 56, 57].

People experiencing homelessness were at higher risk of hospitalisation and mortality from COVID-19 infection than the general population [38, 48, 61, 63]. There was limited high-quality evidence available on these outcomes.

Rates of self-reported adherence to COVID-19 preventive measures were generally high, but qualitative findings revealed the challenges of adherence to measures such as social distancing when living in crowded accommodation [27]. There were no studies on testing rates identified.

There was limited and conflicting evidence on the mental wellbeing of people experiencing homelessness during the pandemic. Some weak evidence suggests that the pandemic has led to increased feelings of social isolation, whereas other evidence indicates improved mental wellbeing in homeless individuals [28, 65].

Inequalities in this population may have widened due to the pandemic, with some evidence showing people experiencing homelessness faced financial issues and problems accessing primary and emergency healthcare services [27, 63].

### Context of Other Literature

The finding of increased risk of COVID-19 infection in European shelters and hotel accommodations is consistent with what has been seen in the United States [16]. This risk is likely related to the known increased transmission of COVID-19 in small, crowded spaces, and households of multiple occupancy [69, 70]. Additionally, infection risk may increase with difficulties adhering to social distancing measures in these accommodation spaces [27].

The possible lower risk for those living on the streets may reflect the reduced spread of COVID-19 in outdoor spaces and areas with better ventilation [71]. However, it is difficult to assess this properly given the transient nature of rough sleepers [72]; there was also no research into how their movement and use of communal spaces may have changed their risk of COVID-19 outcomes.

Increased rates of hospitalisations and mortality in people experiencing homelessness may be consequences of higher rates of infections and baseline worse health. Individuals with poor underlying health and long-term conditions are at greater risk of severe COVID-19 infection and other negative outcomes [12, 73].

Before the pandemic, people experiencing homelessness had extremely high rates of mental health problems [15], but research on the impacts of the pandemic on mental wellbeing is limited and findings conflicting. Evidence on the general population shows that the pandemic has negatively impacted mental wellbeing [74, 75].

Evidence from the UK showed that people experiencing homelessness attended EDs more frequently during the pandemic than the general population [63]. This echoes what is commonly seen in people experiencing homelessness, where their unmet health needs and inability to access services such as primary care lead to high use of emergency services [76].

### Strengths and Limitations of the Evidence Base

Many studies included were low quality, with few (if any) high-quality studies for each outcome, and findings should be interpreted with caution.

Many studies did not define homelessness type, either of their overarching included population or in the analysis. Studies did not document testing and vaccination rates in the populations studied, which are important confounders when looking at clinical outcomes of COVID-19. Testing became commonly available to the general population a few months after the pandemic started (2020). Vaccination became available 12–18 months after the pandemic started (2021) and people experiencing homelessness were not prioritised in several countries. Thus, many earlier studies did not examine this issue. Studies looking at hospitalisation and mortality did not explore differences between subgroups of homeless populations.

Additionally, even research aiming to study all people experiencing homelessness across a given setting struggled to identify all homeless individuals given the difficult nature of researching this population, especially the ‘hidden homeless’ (insecure or inadequate categories of ETHOS, Supplementary Appendix SB).

There was a significant lack of research on how other risk factors interacted with homelessness to affect COVID-19 outcomes. Much evidence has shown that men and Black and ethnic minorities are at higher risk of severe COVID-19 outcomes, but this was not explored in the literature available [77, 78]. Whether impacts were directly due to COVID-19 illness or pandemic measures such as lockdowns cannot be ascertained from this evidence base.

### Strengths and Limitations of the Review

This is the first systematic review on people experiencing homelessness and clinical outcomes and mediators of COVID-19. An overall assessment of quality to highlight studies with good design, methodology, and transferability was used.

Limitations in the methodology of the review include that only 10% of titles and abstracts were screened by two reviewers, and that due to time constraints, data extraction and critical appraisal were conducted by only one reviewer. Additionally, studies not published in English were excluded.

### Implications for Policy and Practice

This review provides evidence that supports concerns regarding the impact of experiencing homelessness on COVID-19 outcomes, and highlights areas of policy and practice that need to be addressed. People experiencing homelessness are often underserved and at higher risk of COVID-19 outcomes such as hospitalisation and mortality. Their vulnerability and inequality in health must be addressed for the current and future stages of this COVID-19 pandemic, as well as for potential future pandemics. Steps should be taken to ensure accommodations follow measures to reduce transmission such as ventilation, social distancing, and single rooms. Initiatives to prioritise and promote vaccination in this population are also vital in preventing further high rates of severe infection and death.

### Implications for Future Research

Research on people experiencing homelessness and COVID-19 remains important to help understanding, practice, and policy. Improvements in the recording of people experiencing homelessness are vital, as research is difficult with much of the population not in national datasets or registered in primary care.

Research gaps relating to COVID-19 outcomes, mediators, and the inequalities in health in this population identified in this review should be addressed. Long-term impacts such as long COVID and the impact on health inequalities in people experiencing homelessness should be explored given the evidence of high rates of long-term conditions and inequality preceding the pandemic. Barriers to vaccination uptake within this population should be further explored, given that it is an important mediator of COVID-19 outcomes.

More research is needed into why hospitalisation and mortality rates are higher and the effectiveness of interventions to improve this. Furthermore, COVID-19 has impacted ethnic minorities more [77], but there is missing evidence on how people from ethnic minorities experiencing homelessness have been impacted specifically.

Differences seen between quantitative and qualitative results indicate that mixed methods approaches may be important in research with people experiencing homelessness to identify and compare findings and investigate reasons for disparities [27].

### Conclusion

This review offers a comprehensive summary of the literature available; however, since 8 of the 41 included papers were high quality, 21 were moderate quality, and 12 were low quality, the overall confidence in the findings is moderate.

The review shows evidence that people experiencing homelessness are at increased risk of COVID-19 outcomes such as infection, hospitalisation, and death. Improvements to accommodation to minimise infection, management of long-term physical and mental health, and reducing inequalities of access in general to healthcare—and barriers towards vaccination in particular—are important for this vulnerable population. Further research is needed to understand and prepare for the long-term implications that may arise in people experiencing homelessness, both in further waves of COVID-19 and potential further pandemics.
