# Supplementary material for: The Impact of Being Homeless on the Clinical Outcomes of COVID-19: Systematic Review
Source: Int J Public Health. 2023 Sep 15;68:1605893. doi: 10.3389/ijph.2023.1605893 (PMC10540688; doi:10.3389/ijph.2023.1605893)
Supplement: Supplementary file 1 [file DataSheet2.pdf]

Appendix B-Abbreviated ETHOS framework of homelessness and housing exclusion

Table 1. Abbreviated ETHOS framework of homelessness and housing exclusion (20)

	Operational Category	Living Situation
**Roofless**	1. People Living Rough	Public or external space
	2. People in emergency accommodation	Night shelter
**Houseless**	3. People in accommodation for homeless	Hostel, temporary or transitional accommodation
	4. People in Women's Shelter	Women's shelter
	5. People in accommodation for immigrants	Temporary or migrant workers accommodation
	6. People due to be released from institutions	Penal, medical or children's institutions
**Insecure**	7. People receiving longer-term support (due to homelessness)	Residential care for older homeless people
	8. People living in insecure accommodation	Temporarily with family/friends, no legal tenancy, illegal occupation
	9. People living under threat of eviction	Legal or re-possession orders
	10. People living under threat of violence	Police recorded incidents
**Inadequate**	11. People living in temporary/non-conventional structures	Mobile homes, non-conventional buildings, temporary structure
	12. People in unfit housing	Occupied dwellings unfit for habitation
	13. People living in extreme overcrowding	Highest national norm of overcrowding
