# Supplementary material for: The Impact of Being Homeless on the Clinical Outcomes of COVID-19: Systematic Review
Source: Int J Public Health. 2023 Sep 15;68:1605893. doi: 10.3389/ijph.2023.1605893 (PMC10540688; doi:10.3389/ijph.2023.1605893)
Supplement: Supplementary file 2 [file DataSheet1.pdf]

Appendix A: Search Strategy of databases

Medline via OVID 28.11.22

	String line	Number of results
1	exp Coronavirus/	160423
2	COVID-19/	210509
3	((corona* or corono*) adj1 (virus* or viral* or virinae*)).ti,ab,kw	5338
4	(coronavirus* or coronovirus* or coronaviri* or 2019-nCoV or 2019nCoV or nCoV2019 or nCoV-2019 or covid-19* or covid19* or ncov* or n-cov* or HCoV* or SARS-CoV-2 or SARSCoV-2 or SARSCov2 or SARS-CoV2 or severe acute respiratory syndrome).ti,ab,kw	341577
5	((outbreak* or pandemic* or epidemic*) adj10 (wuhan or hubei or china or Chinese or Huanan)).ti,ab,kw	11698
6	1 or 2 or 3 or 4 or 5	359003
7	exp homeless persons/ or exp homeless man/ or exp homeless youth/ or exp homeless woman	10711
8	(homeless* or unhouse* or unshelter* or roofless* or houseless* or "sleeping on the street*" or "living on the street*" or "sleeping rough" or "living rough" or "rough sleep*" or "street person*" or "street people" or "street liv*" or "without a roof").ti,ab.	13499
9	("no fixed address*" or "no fixed abode" or "unstable hous*" or "hous* instability" or "lack of hous*" or "vulnerably hous*" or "night shelter" or "transition hous*" or "supported hous*" or "emergency hous*" or "emergency shelter*" or "temporary accommodation" or "emergency accommodation" or "insecure accommodation" or "precarious hous*" or "seeking shelter" or "couch surf*" or "sofa surf*").ti,ab	2225
10	(street dwell* or improvised dwell* or shelter dwell* or sleeping out* or street involved).ti,ab	301
11	((without or no or "lack of" or inadequate*) adj1 (hous* or accommodation* or shelter* or hostel* or dwell*)).ti,ab	1819
12	((homeless* or street or transient* or marginal* or vulnerabl* or temporary or unstabl* or vulnerabl* or insecure or support* or transition*) adj2 (hous* or accomondation* or shelter* or hostel* or dwell*)).ti,ab.	5687
13	7 or 8 or 9 or 10 or 11 or 12	21709
14	6 and 13	949
